# New Method for the Deposition of Nickel Oxide in Porous Scaffolds for Electrodes in Solid Oxide Fuel Cells and Electrolyzers

**DOI:** 10.1002/cssc.201600813

**Published:** 2016-10-14

**Authors:** Enrique Ruiz‐Trejo, Milla Puolamaa, Brian Sum, Farid Tariq, Vladimir Yufit, Nigel P. Brandon

**Affiliations:** ^1^ Department of Earth Science and EngineeringImperial College London InstitutionLondonSW7 2AZUnited Kingdom

**Keywords:** ceramics, electrochemistry, fuel cells, nickel, surface analysis

## Abstract

A simple chemical bath deposition is used to coat a complex porous ceramic scaffold with a conformal Ni layer. The resulting composite is used as a solid oxide fuel cell electrode, and its electrochemical response is measured in humidified hydrogen. X‐ray tomography is used to determine the microstructural characteristics of the uncoated and Ni‐coated porous structure, which include the surface area to total volume, the radial pore size, and the size of the necks between the pores.

## Introduction

The use of metal infiltration into a ceramic scaffold to fabricate electrodes for solid oxide fuel cells and electrolyzers (SOFC/SOEC) has been demonstrated successfully in terms of electrochemical performance.^1^ The concept is to first fabricate a ceramic scaffold for the electrolyte and then deposit NiO by infiltration. During infiltration, the pores are saturated with a metal nitrate solution and then treated thermally at 500–700 °C to decompose the solution into the metal oxide. The incorporation of two electrode components, for example, BaCe_0.5_Zr_0.3_Y_0.16_Ni_0.04_O_3−*δ*_ (BCZYZ) and Ni, at different processing stages means that the microstructure of the electrode can be controlled independently for each component.^2^ The electrode is expected to be more tolerant to redox‐cycling as the volume changes experienced by Ni can be accommodated by the porous structure.^3^ Infiltration, also known as impregnation, yields excellent results for Ni/Gd‐doped ceria and for Ni/BCZYZ in terms of Ni distribution^2^, ^4^ and low polarization resistance.^4^ Nonetheless, infiltration is a time‐ and energyconsuming technique and its scale‐up is problematic. Furthermore, the instability of the infiltrated nanostructure needs to be addressed as initial work indicates that the infiltrated material can degrade rapidly depending upon the number of infiltration cycles.^5^

The most common method to coat a substrate with Ni is electroless deposition from an aqueous Ni solution using a strong reducing agent such as H_2_NNH_2_, NaPO_2_H_2_, or NaBH_4_.^6^ There are several examples of this method applied to SOFC anodes.^7^ Unfortunately, hydrazine is highly toxic and the other reducing agents leave P or B residues in the deposit that are detrimental to anode performance; furthermore, expensive activating agents are necessary, usually based on Pd, which adds costs and complexity to the process.7g Other alternatives for Ni incorporation have been tested, which include microwave‐assisted infiltration^8^ and a combination of Ag electroless and subsequent Ni electrodeposition.^9^ The challenge addressed in this work is to explore a new method of Ni deposition without compromising distribution and performance. In this study we use a simple chemical bath solution to deposit NiO. The chemical bath deposition of NiO has been used for optical devices and supercapacitors,^10^ but to the best of our knowledge, this work is the first study to utilize a similar approach to fabricate SOFC/SOEC electrodes by the deposition of Ni in a highly complex porous structure. In addition to process development and materials characterization, a second objective is to use state‐of‐the‐art X‐ray microtomography to determine the microstructural properties of the scaffold and to understand the nature of the Ni deposit, the conditions for its optimal incorporation, and its influence in the fabrication process.

## Results and Discussion

The precipitation, microstructure, and reduction of Ni in a BCZYZ scaffold are addressed first. The initial chemical bath solution was deep blue, and the precipitate obtained was black and easily distinguishable by eye. The deposition onset varied between 1 and 60 min. The times displayed in the following text correspond to the point at which precipitate was observed. The precipitate consisted of a mixture of nickel oxy‐hydroxides and they will be referred to as NiO_*x*_.

**Figure 1:**
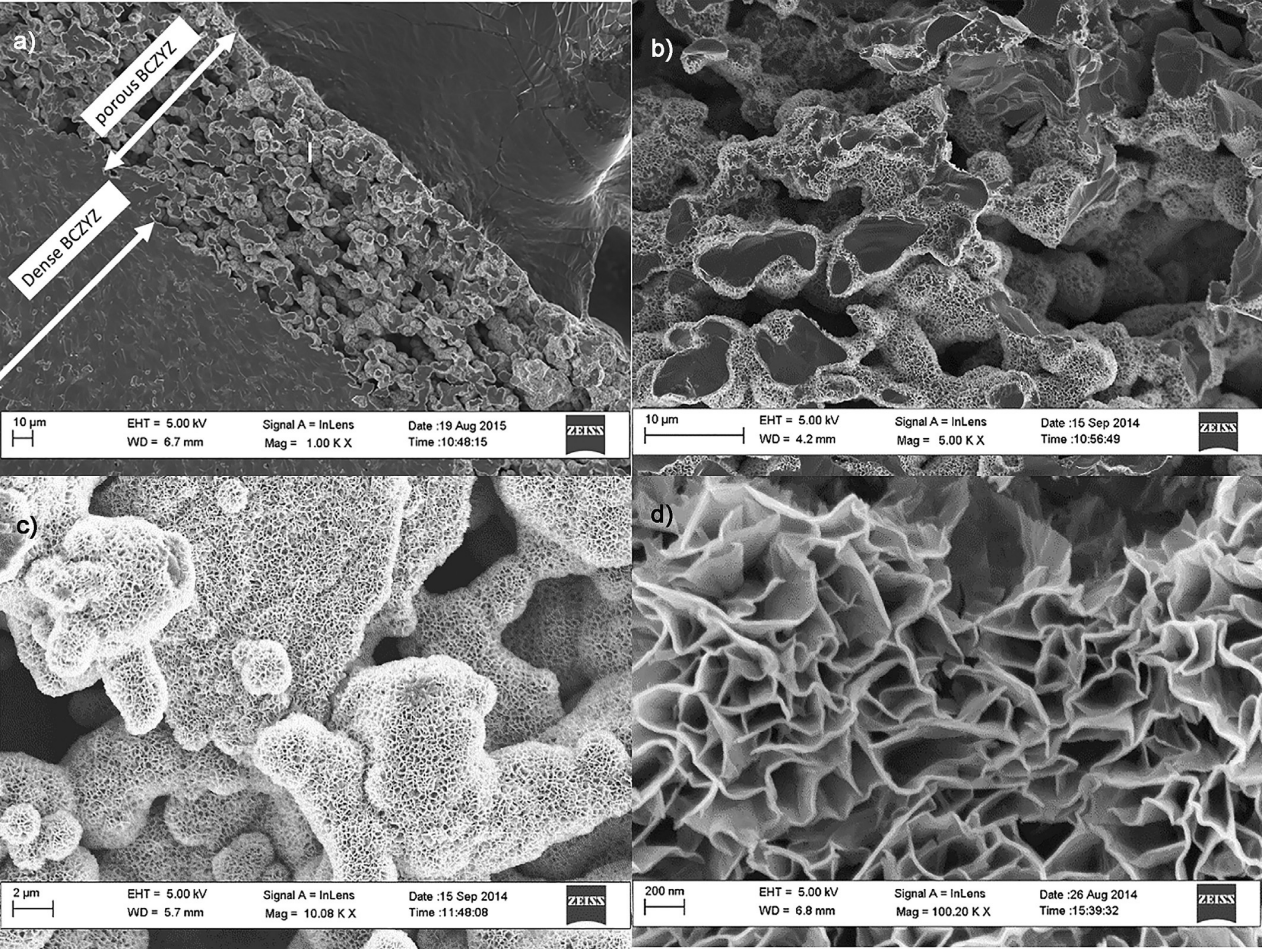
Micrographs that show fracture surfaces of cross‐sections of the BCZYZ wafers. a) Cross‐section that shows the overall dimensions of the scaffolds and the homogenous distribution of NiO_*x*_. b) High‐magnification image that shows the NiO_*x*_ deposits around fresh fractured surfaces. c) Reticular structure of the NiO_*x*_ precipitate. d) Magnification of 100 000× that shows very thin (≈30 nm thick) platelets that form a continuous network.

The NiO_*x*_ deposit was black and had an excellent adhesion to the scaffold without the need to sensitize and activate the surface, as it is often the case during the electroless coating of Ni.^6^, 7e Four SEM images of fractured BCZYZ wafers that show a continuous and homogeneous coating of NiO_*x*_ are shown in Figure 1 a–d. The high‐magnification micrograph revealed that the Ni precipitate was formed of fine platelets with a thickness of approximately 30 nm separated from each other by several hundred nanometers to form a reticular structure, in agreement with previous studies.10a, 10b

**Figure 2:**
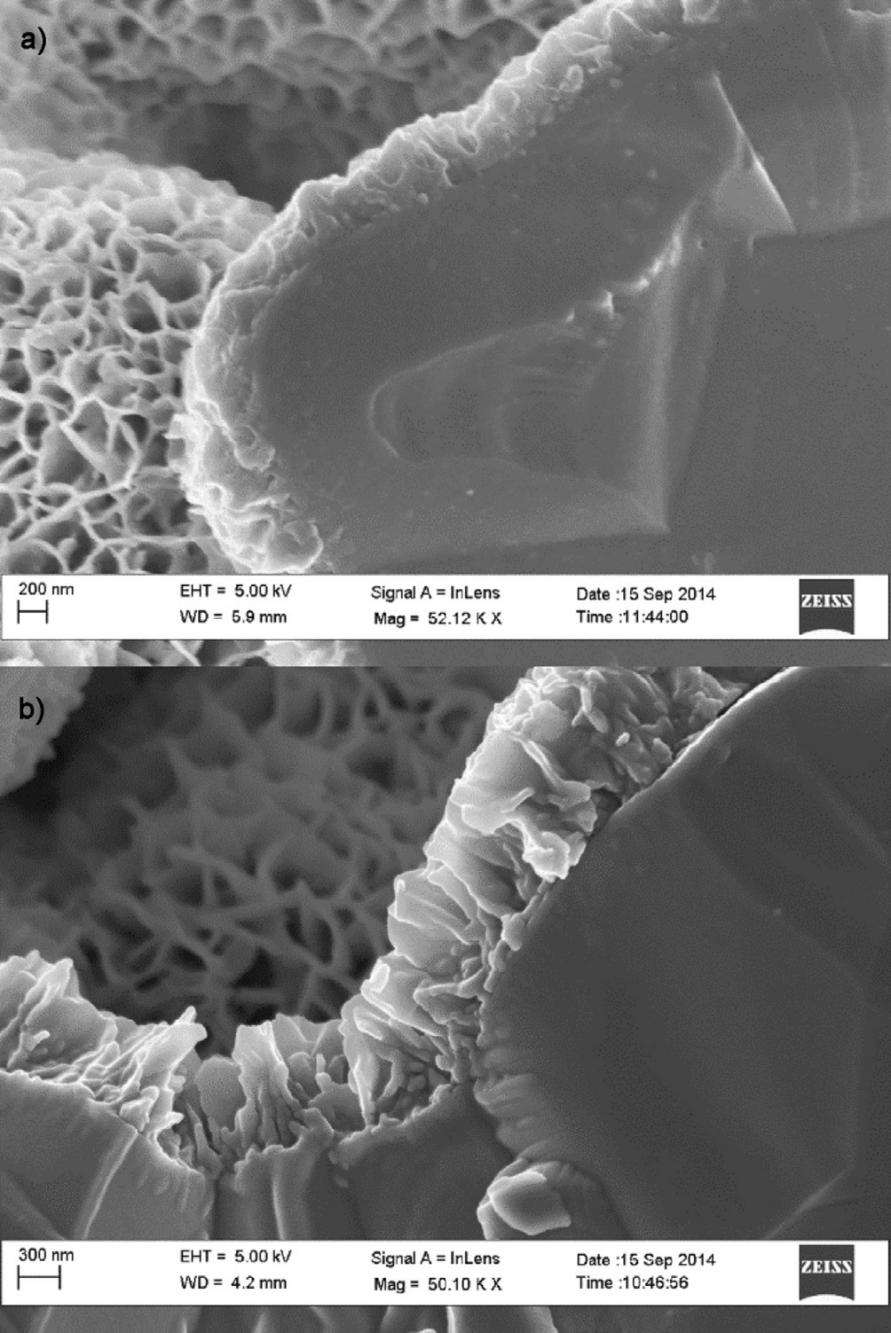
Cross‐section of NiO_*x*_ deposits on BCZYZ with a) a deposition time of 60 min and a thickness of ≈0.5 μm and b) a deposition time of 120 min and a thickness of ≈0.95 μm.

During the early stages of this work, it was observed that the nickel oxide did not penetrate into the scaffold uniformly unless the solution was stirred gently. Uniform coatings of the nickel oxide were achieved as seen in Figure 1 a and later confirmed by tomography. According to previous studies,10b this reticular microstructure is retained at temperatures up to 300 °C at which all the hydroxides are eliminated completely and only nickel oxide remains. Micrographs after 1 and 2 h deposition time are shown in Figure 2. The thickness of the nickel oxide deposit inside the pores grew at a rate of around 0.5 μm h^−1^ over the first 2 h of deposition.

In baths left overnight, the deposit grew up to 1 mm on top of the scaffold, but the SEM images (not shown) did not show any noticeable increase in the amount of Ni inside the pores; this indicated clearly that the Ni concentration inside the pores was not sufficient to sustain the layer growth and that the diffusion of Ni ions from the bulk of the solution was impeded as a result of the growth of the thick layer on top of the scaffold.

**Figure 3:**
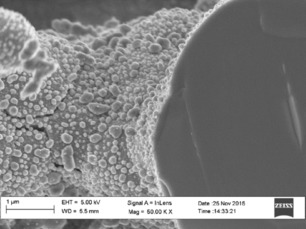
Cross‐section of fresh fractures (scale bar corresponds to 1 μm) that shows the reduced Ni deposited by using the chemical bath for 30 min and then reduced at 500 °C for 1 h in 10 % H_2_+3 % H_2_O+87 % N_2_.

Upon reduction, the microstructure of the infiltrated nickel oxide changed dramatically: the original deposit was clearly interconnected but upon heating and reduction, the structure was transformed to metal droplets of 20–130 nm in diameter (Figure 3). The resulting structure of Ni is similar to that reported on a Gd‐doped CeO_2_ scaffold.4a Although the structure shown is not percolating, the incorporation of Ni into the scaffold by chemical bath deposition is significantly faster than infiltration as it only took 30 min to deposit a layer of NiO_*x*_ compared to at least 2.5 h needed for the infiltration in addition to cooling and heating times.

**Figure 4:**
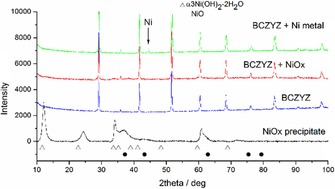
XRD pattern of the Ni precipitate, uncoated BCZYZ, BCZYZ coated with the Ni precipitate, and BCZYZ after the reduction of the precipitate to Ni metal. The expected NiO reflections are indicated with dark circles, the nickel hydroxide with empty triangles, and the only clear reflection of Ni metal is indicated with an arrow at 2 *θ*=44.6°.

The XRD pattern of the NiO_*x*_ precipitate collected by filtration from the solution is shown in Figure 4. The NiO_*x*_ precipitate was dried at 70 °C for 1 h before the XRD measurement and it consisted of a mixture of nickel oxy‐hydroxides. The exact position and intensity of the precipitate reflection peaks varies slightly to that described previously,10a, 10b, 11 which is probably associated with the amount of water in the structure and the synthesis conditions. As a reference, the reflections of NiO and α‐3 Ni(OH)_2_⋅2 H_2_O (JCPDS file: 22‐044) are indicated in the plot. For the purpose of this work, the exact stoichiometry of the precipitate is not relevant as it is decomposed eventually at high temperatures (*T*>300 °C) to NiO and reduced ultimately to Ni metal under hydrogen (*T*>350–400 °C). The XRD patterns of the uncoated and coated BCZYZ wafers are also shown in Figure 4. The NiO_*x*_ is barely noticeable because of the thin nature of the layer and the low crystallinity of the deposit. Finally, upon reduction a peak at 2 *θ*=44.6° shows the presence of Ni metal unambiguously. Further confirmation of the presence of Ni metal was obtained by attracting the sample with a strong magnet.

**Figure 5:**
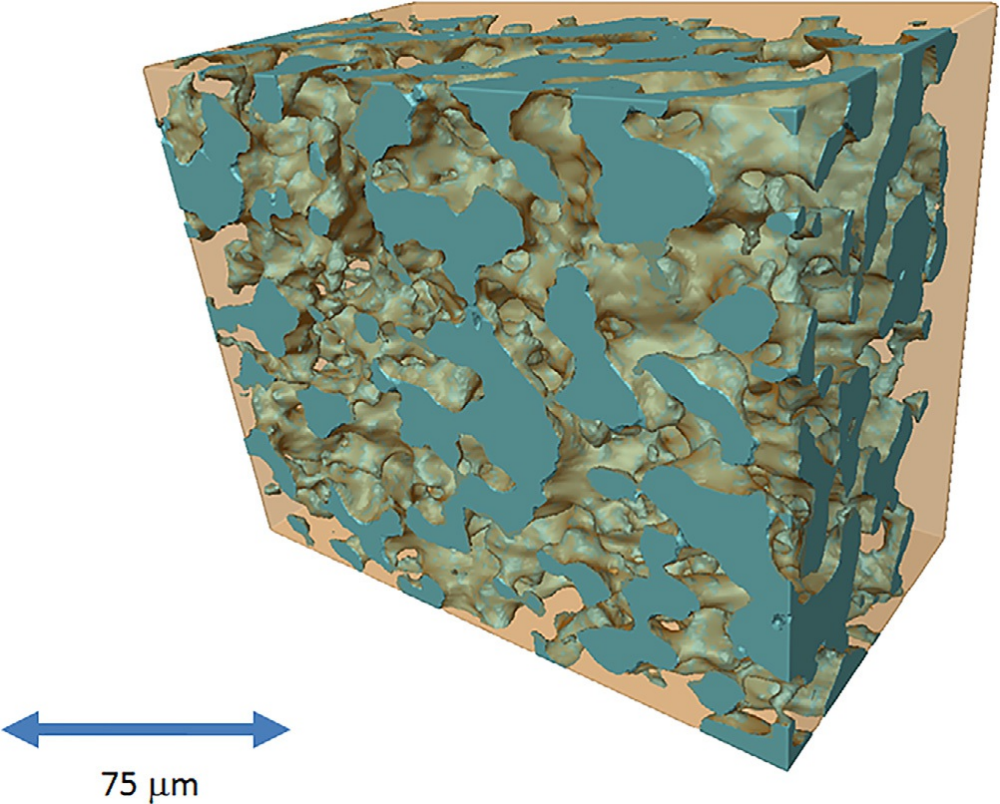
Uncoated BCZYZ scaffold highlighting the porous network.

An uncoated scaffold and one deposited with NiO_*x*_ for 2 h were scanned by using the X‐ray microtomography system, and their 3 D microstructures were obtained. The reconstructed 3 D image of an uncoated BCZYZ scaffold is shown in Figure 5. A video is given in the Supporting Information that displays the complex 3 D microstructure dynamically. The results reveal a very well‐connected path of open porosity and negligible closed porosity at this resolution. A summary of the extracted microstructural parameters is shown in Table 1. The scaffold had a porosity of 36 vol % and a volume specific surface area of ≈0.14 μm^2^ μm^−3^. The data for a sample coated with NiO_*x*_ by using the chemical bath technique for 2 h are displayed in Table 2. There was a small increase in the volume specific surface area to 0.17 μm^2^ μm^−3^ for the NiO_*x*_‐coated scaffold. This is probably a consequence of the NiO_*x*_ deposit and the textured structure that it creates, as revealed by SEM imaging (Figure 1).

**Table 1:** Microstructural data obtained for an uncoated BCZYZ scaffold

Structure	Area [μm^2^]	Volume [μm^3^]	Volume [%]	Surface area/volume for pores [μm^2^ μm^−3^]	Surface area/total volume [μm^2^ μm^−3^]
pores	1.12×10^6^	2.85×10^6^	36	0.39	0.14
bulk	1.25×10^6^	5.15×10^6^	64	0.24	0.16

**Table 2:** Microstructural data for a nickel oxide deposited BCZYZ scaffold.

Structure	Area [μm^2^]	Volume [μm^3^]	Volume [%]	Surface area/volume for pores [μm^2^ μm^−3^]	Surface area/total volume [μm^2^ μm^−3^]
pores	0.47×10^6^	1.02×10^6^	37	0.462	0.171
bulk	0.51×10^6^	1.70×10^6^	62	0.298	0.184

**Figure 6:**
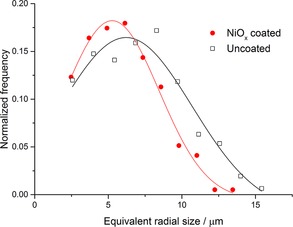
Normalized frequency versus pore size for the uncoated and coated BCZYZ scaffold. A Gaussian fit was used to determine the median of the peaks as 6.21 and 5.26 μm for the uncoated and coated scaffolds, respectively.

Quantification of the equivalent spherical radial pore sizes (Figure 6) shows a subtle change between the uncoated and coated samples. The modal radial pore size decreased by 0.95 μm following NiO_*x*_ coating. Similarly, the pore sizes after coating were all decreased across the entire range for which there is data, with a shift in the same order of size as the modal peak sizes. These values, which consider thousands of pores, are insightful as the change corresponds to the thickness of the coating, and the estimated growth rate of 0.455 μm h^−1^ is consistent with the thicknesses observed in the image displayed in Figure 2.

The surface area to total volume ratio in given in Tables 1 and 2 can be compared with that of a ≈10 μm thick screen‐printed Ce_0.9_Gd_0.1_O_2−*x*_ scaffold.^2^ Although the materials and processing methods are different, both scaffolds need to incorporate Ni to work as anodes. The area of the solid phase per unit volume in Ce_0.9_Gd_0.1_O_2−*x*_ is 6.44 μm^2^ μm^−3^ as determined by using focused ion beam scanning electron microscopy (FIB‐SEM), whereas that of the tape‐cast scaffold of BCZYZ is 0.14 μm^2^ μm^−3^. The area is 54 times larger for Ce_0.9_Gd_0.1_O_2−*x*_ and will, therefore, have a larger area per unit volume available for the formation of triple phase boundaries upon Ni incorporation.

**Figure 7:**
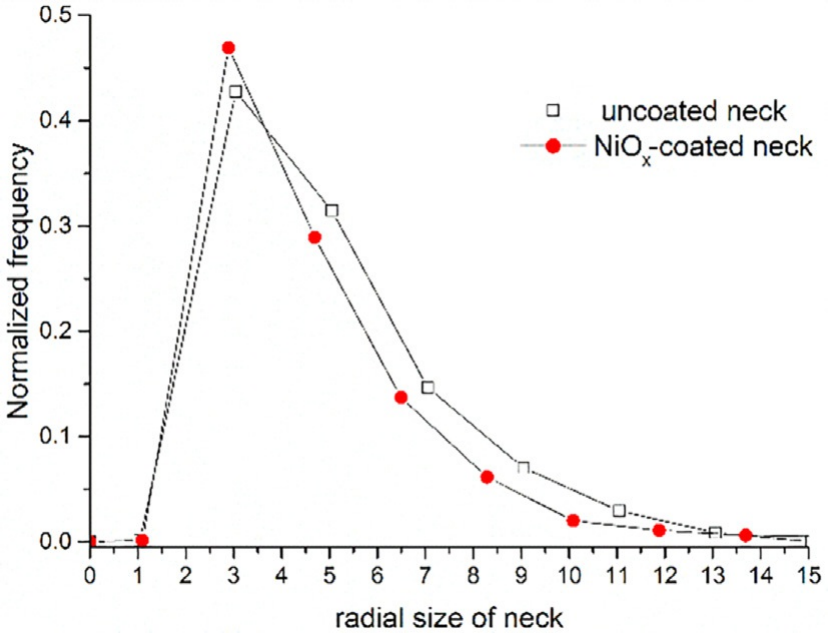
Normalized frequency versus size of necks for uncoated and NiO_*x*_‐coated porous BCZYZ.

It was also possible to calculate and subsequently quantify necks in the electrode. The necks are defined as the cross‐sectional surface (constriction) between two adjacent particles of the same phase, that is, pores in this case.^12^ A video is given in the Supporting Information that displays the necks in the sample dynamically. The normalized frequency against neck radius for uncoated and NiO_*x*_‐coated scaffolds is given in Figure 7. The majority of the radii of the necks for the coated sample have ≈3 μm equivalent circular sizes. As in the case of the pores, the estimated shift value between the fitted peak maxima is 0.9 μm. This value is consistent with the thickness of the deposit, obtained by the analysis of both the pore radial size and the SEM images. This quantity has consequences for the coating method as an effective deposition process requires the continuous diffusion of active Ni ions from the solution bulk to the pore network. One of the factors that affects the flow of fluids inside the structure is not only the size of the pores but also the size of the necks. A rough growth rate for the NiO_*x*_ layer was estimated to be 0.95 μm in 2 h according to Figure 6, and the implication is that most common necks will be blocked after 3 h of continuous deposition. This explains why, even after it is left overnight in the chemical bath, the scaffold was not filled completely with the Ni precipitate yet the uppermost surface of the scaffold had a thick deposit.

**Figure 8:**
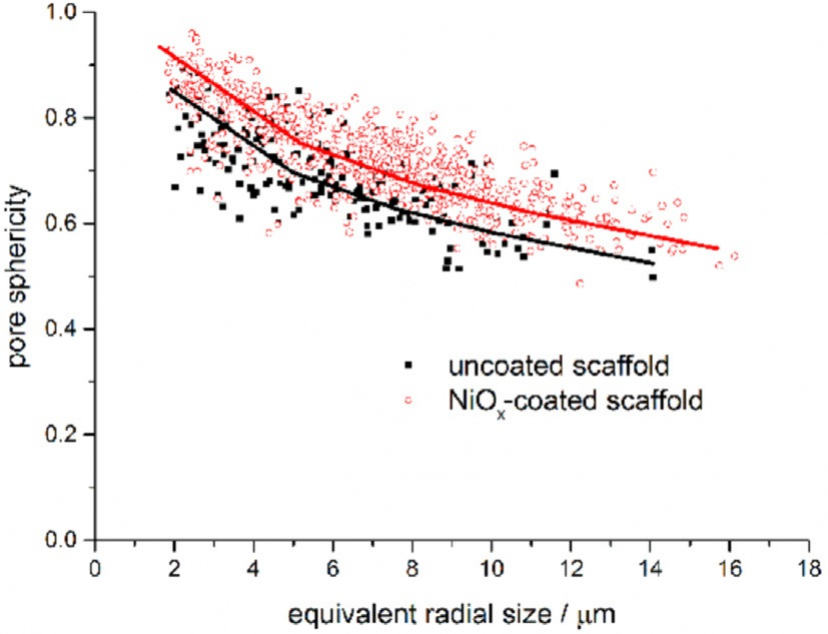
Pore sphericity/roundness for uncoated and Ni‐coated porous BCZYZ backbone (perfect sphere=1). The lines are a guide to the eye.

An interesting microstructural feature of the scaffolds is the sphericity of the pores (in which sphericity is defined as the ratio of a perfect sphere of volume *V* and the area of the actual pore with the same volume *V*). The maximum normalized frequency is at 6.2 μm and, if the 0.95 μm coat thickness is relatively constant all over the sample, a change in the sphericity of the pores is not expected (Figure 6). The pore sphericity for the coated and uncoated samples is shown in Figure 8. This shows that they differ only slightly, which indicates a homogeneous coating. It is also clear that small pores are more spherical that larger ones, which may be related to the pore formers used for the tape‐casting, namely, glassy carbon (spheres of 1–6 μm radius) in addition to graphite flakes^12^ (platelets of >10 μm length).

The roundness of the samples is shown in Figure 8, and the roundness is based on a hypersphere fit around a given pore and its subsequent deviation from it. A perfect sphere would have a value of 1, and elongated rough pores would have lower values. It was found for both coated and uncoated samples that as pore sizes increase their roundness decreases. However, a small increase in the roundness of the pores occurred upon coating. Given the similarity of both scaffolds shown in Figure 8, the results show consistent levels of Ni deposition across all pore sizes in this electrode. No significant deposition occurs at a particular pore size that affects its shape strongly, which indicates a homogenous deposit.

### Electrochemical performance

Two samples with similar Ni loadings but prepared by different methods were compared. A sample with 6 wt % Ni metal prepared by the established infiltration method (In‐3x) and a sample with 7 wt % Ni made by the chemical bath method (Cb‐2x) were analyzed by using electrochemical impedance spectroscopy (EIS) in a symmetrical cell arrangement in 97 % H_2_+3 % H_2_O.

**Figure 9:**
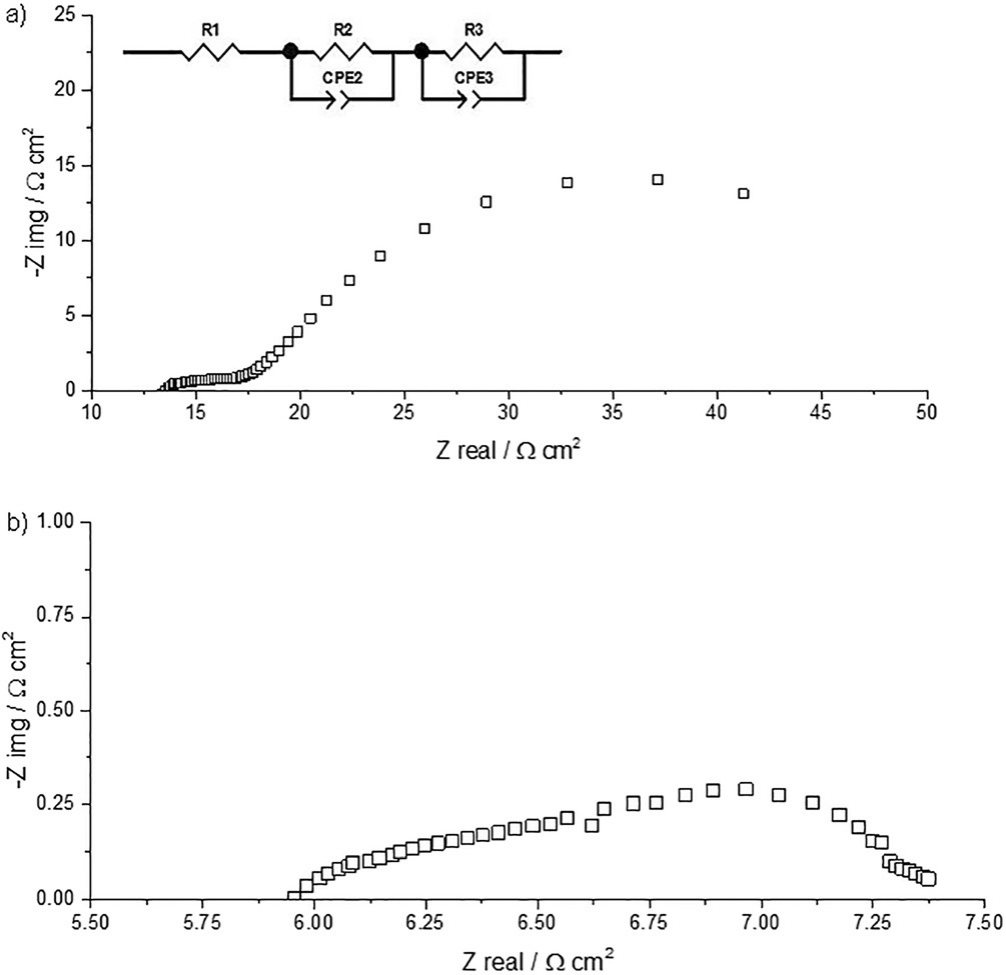
Impedance spectrum in 97 % H_2_+3 % H_2_O at a) 500 and b) 800 °C. CPE=constant phase element.

Nyquist plots for Cb‐2x at 500 and 800 °C are shown in Figure 9. The raw impedance values were multiplied by the geometrical area of one electrode and divided by two to normalize the data. The impedance spectra were similar in shape at both temperatures and showed a low‐frequency (LF) and intermediate‐frequency (IF) semicircle. The normalized data were fitted with the equivalent circuit shown in the inset of Figure 9 a, in which *R*1 corresponds to the Ohmic resistance and *R*2 and *R*3 to the processes at intermediate and low frequency, respectively. These normalized values are area‐specific resistances (ASR).

**Figure 10:**
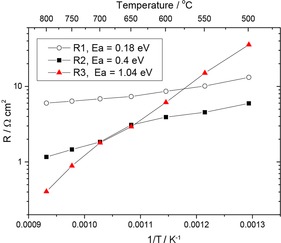
ASR of a symmetrical Ni‐BCSCZ cell as a function of temperature estimated from fitting the data to the equivalent circuit shown in Figure 9 a.

The area‐specific resistances and associated activation energies for *R*1–*R*3 are shown in Figure 10. Clearly, *R*1 is associated with the conductivity of the dense BCZYZ electrolyte: at 600 °C its value was estimated to be 1.1 mS cm^−1^ (compared with 2.1 mS cm^−1^ reported previously at this temperature^13^); the activation energy was 0.18 eV, similar to the reported values of 0.22 eV for BaZr_0.9_Y_0.1_O_3−*δ*_,^14^ but in particular it is closer to the activation energy of hydrogen bulk diffusivity of 0.13 and 0.25 eV for BaZr_1−*x*_Y_*x*_O_3−*δ*_ with *x*=0.1 and 0.2, respectively, measured by using quasielastic neutron scattering.^15^ From the data presented in Figure 10 it is also clear that most of the losses are because of electrolyte resistance (the thickness in this sample was 192 μm), except at temperatures <550 °C at which *R*3 dominates.

The third element of the impedance spectra, *R*3, at the lowest‐frequency values, is most likely associated with the dissolution or diffusion of hydrogen in Ni and Ag (used as a current collector). *R*3 has an activation energy that is similar in behavior to Ni/Ag–proton conductor (Sr_0.995_Ce_0.95_Y_0.05_O_2.975_) interfaces,^16^ for which impedance measurements revealed that the Ag electrode had a higher activation energy (0.93 eV) than the Ni electrode (0.84 eV) and that these values were consistent with the activation energy of the cathodic limiting current of 1.1 eV for Ag and 0.70 eV for Ni. The authors concluded that the rate‐limiting behavior in the electrodes was possibly because of the nondissociative adsorption or diffusion of hydrogen in the metal phase.^16^, ^17^ From the data shown in Figure 10 at 600 °C, *R*3 (LF semicircle: 0.1–1 Hz) has an associated resistance of 6.1 Ω cm^2^. The activation energy is 1.04 eV, which leads to small polarization resistances of 0.45 Ω cm^2^ at 800 °C. The LF contribution is probably associated with the diffusion of hydrogen in the metal components of the cell.

The identification of *R*2 in the IF range is not as straightforward as that of the other two elements and can only be assumed to be a mixture of several factors, among them the charge transfer reaction. A total electrode ASR at 800 °C (i.e., *R*2+*R*3) is ≈1 Ω cm^2^. An overall comparison between the infiltrated and the chemical bath sample at 600 °C provided a better evaluation of the two methods.

**Figure 11:**
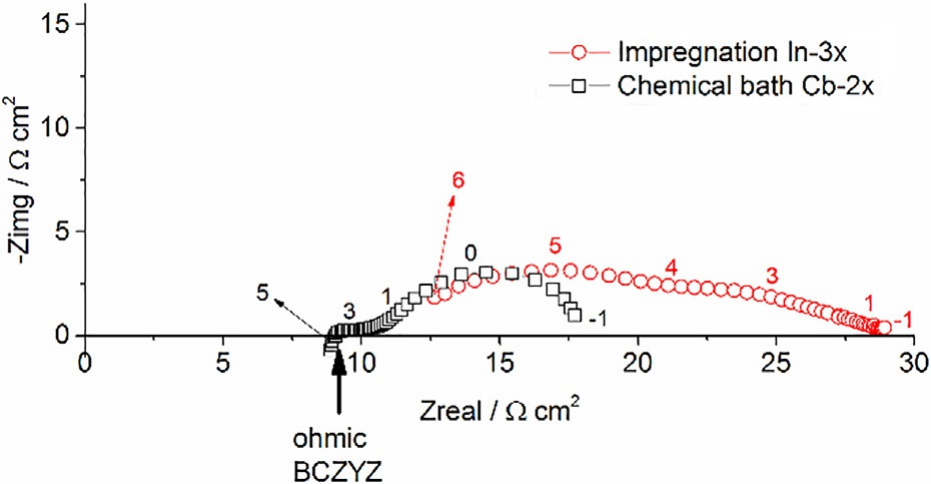
Impedance plot for infiltrated and chemical bath deposits measured at 600 °C in 97 % H_2_+3 % H_2_O. The arrow indicates the expected Ohmic resistance for a BCZYZ electrolyte with 200 μm thickness. The values next to the data indicate the log_10_ of the frequency.

The impedance plots of In‐3x and Cb‐2x are presented in Figure 11. As Ag was used in both cases as a current collector, it is assumed that its contribution to the LF polarization is similar in both samples. We see that the Ohmic resistance expected from the electrolyte (≈9 Ω cm^2^) is relatively similar in both cases, although slightly larger in the infiltrated sample. Therefore, if we compare *R*2+*R*3, the electrode fabricated by chemical bath deposition showed a better performance (i.e., a smaller value for *R*2+*R*3).

Further comparison at higher temperatures was not possible as the infiltrated sample response deteriorated rapidly, which indicates that the microstructure obtained by both methods leads to two different rates of degradation. Notably, a higher metal content is necessary to address issues of stability and electrochemical activity, yet these initial measurements were intended to provide a preliminary evaluation of the chemical bath deposition. A rapid degradation in insufficiently infiltrated scaffolds has been observed before^5^ and has been assigned to lost percolation between Ni particles.

**Figure 12:**
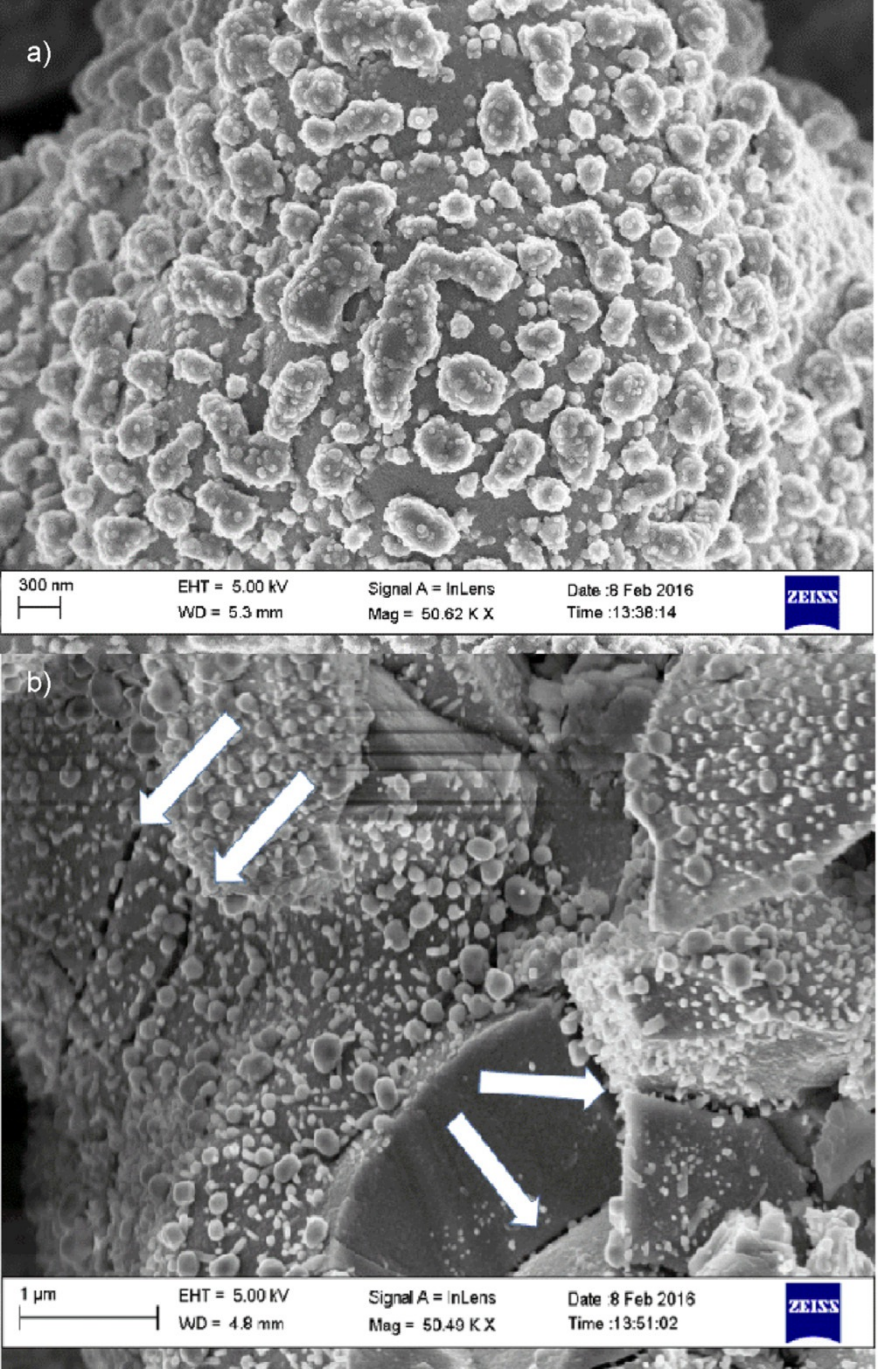
Postmortem micrographs of samples after measurement at temperatures up to 750 °C in humidified hydrogen. a) CB‐2x and b) In‐3x, which shows visible fractures (see arrows).

The microstructure of the two electrodes after impedance measurements is compared in Figure 12. The first clear observation is the loss of percolation, which is expected because of the low levels of Ni. The second relevant observation is the clear structural damage in the infiltrated sample with visible fractures, which were not seen in the chemical bath sample. Both samples were heated above 750 °C and probably experienced Ni agglomeration and the consequent loss of percolation as the contents of Ni are small. There are reasons to assume that damage is not produced during the measurement but during the process of Ni incorporation: the chemical bath technique is performed at room temperature, but the infiltration process requires repeated heating and cooling from room temperature to 500 °C. It is likely that the heating rates used for infiltration (≈10 °C min^−1^) were too high for the sample and caused the cracks seen in Figure 12 b. Additionally, during the decomposition of the nitrates, the corrosive NO_*x*_ produced can react with a basic oxide such as BCZYZ.

Finally, a suggested procedure to obtain a complete fuel cell is to sinter (1300 °C) a wafer of BCZYZ that consists of two tape‐cast layers: a porous BCZYZ and a dense BCZYZ. A cathode can then be screen‐printed and sintered (1000–1150 °C) on top of the dense layer. Finally, after masking the cathode (with a nitrocellulose‐based lacquer) the Ni can be deposited on the porous scaffold using the chemical bath presented in this work. As this last incorporation takes place at room temperature, many other metals that cannot stand the high temperatures required to sinter BCZYZ can be incorporated easily by using similar chemical baths or other low‐temperature methods.

## Conclusions

It was demonstrated that complex shapes can be coated evenly with nickel oxide without the need for sensitization or activation of the BaCe_0.5_Zr_0.3_Y_0.16_Ni_0.04_O_3−*δ*_ surface by using a simple chemical bath. The initial reticular NiO_*x*_ structure grew at a rate of ≈0.445 μm h^−1^ in a porous scaffold. The reticule collapses if heated but it remains attached to the scaffold structure and has been used to fabricate a solid oxide fuel cell anode. A 7 w % Ni metal content was enough to produce an area‐specific resistance of ≈1 Ω cm^2^ at 800 °C, and in general, the total area‐specific resistance was smaller than that of an anode fabricated by infiltration with a similar Ni content.

X‐ray tomography was used to study the scaffold before and after deposition and it was shown that the deposition method presented here was limited only by the nature of the scaffold structure: after 3 h of deposition the interconnecting necks of 3 μm simply block the flow of the chemical bath solution, which impedes further deposition. Furthermore, we demonstrated that the tomography technique can be used to estimate useful information for the scaffold such as the surface area to total volume ratio, porosity percentage, and the thickness of the deposit.

## Experimental Section

### Scaffolds

The ceramic electrolyte selected for the study was a high‐temperature proton‐conducting perovskite BaCe_0.5_Zr_0.3_Y_0.16_Zn_0.04_O_3−*δ*_ used for fuel cells and CO_2_ electrolysis.4b, 13 This material was synthesized and processed as reported previously^13^ to produce three‐layer wafers with a porous/dense/porous configuration with a thickness of approximately 100/200/100 μm, respectively.

### Chemical bath deposition

Typically, nickel sulfate hexahydrate (4 mL, 1 m, ACS reagent, 99 %, Aldrich) was mixed with ammonium persulfate (3 mL, 0.25 m, ACS reagent, ≥98.0 %, Aldrich) and concentrated aqueous ammonia (1 mL, 28 %, Aldrich). The mixture was then topped up with deionized water up to a total volume of 10 mL. The BCZYZ wafer was suspended in a vial with the stirred solution at RT and held for different times (0.5–12 h). Once coated, the samples were rinsed thoroughly with deionized water to eliminate all soluble salts and dried at 70 °C. In the indicated cases the deposition procedure was repeated twice. After deposition, the samples were reduced at 500 °C for 1 h in 10 % H_2_+90 % N_2_ unless otherwise indicated. To test the presence of Ni metal, samples were placed close to a strong magnet; for percolation, samples were tested for continuity by using a digital multimeter. FEG‐SEM (Leo 1550) was used to image the microstructure immediately after deposition, reduction, and impedance measurements. XRD diffraction (X′Pert PRO MRD X‐Ray) was used for phase identification at RT.

### X‐ray tomography

Uncoated and NiO_*x*_‐coated (2 h) scaffolds were analyzed. The infiltrated electrode was mounted in an X‐ray computed tomography system (Phoenix Nanotom s, GE, USA) for direct imaging/tomography at 110 kV. The data were acquired at 595 nm (uncoated) and 596 nm (coated) voxel size with at least 3600 projections acquired over a range of 360°. Following reconstruction, a 3 D medial filter (3×3×3 voxel kernel) was used to reduce small quantities of noise in the dataset. The microstructure was segmented by intensity‐based thresholding to separate the different phases present (bulk and porosity). A marching cube algorithm was then used to calculate both the surface area and volume of the porosity, which provided the surface roughness ratios. For further details on the method and its use, the reader is kindly referred to studies published previously.^12^, ^18^ Subsequent segmentation was performed manually using Avizo Fire (FEI, Bordeaux, France) to separate regions and features of interest in the 3 D tomographic datasets as detailed previously.^19^ Advanced quantification was performed using QuiQ 3 D software (IQM Elements, London, UK).

### Electrochemical performance

To determine the electrochemical performance of the anode manufactured by the chemical bath, two symmetrical cell samples were prepared. A sample was immersed in the chemical bath for 2 h under constant stirring, then its surface was wiped with a soft sponge, and it was immersed again for 2 h. The sample was rinsed and dried. The NiO loading was determined by weight change before and after deposition. This sample was labeled Cb‐2x. A second sample was infiltrated with a 2 m Ni(NO_3_)_2_ ethanolic solution and then heated to 500 °C. This process was repeated three times in total, and this sample was labeled In‐3x and was used for comparison. The Ni metal content in In‐3x was 6 wt % and that in Cb‐2x was 7 wt %. Both samples were analyzed at 600–800 °C by using EIS (Autolab PGSTAT 302) in a symmetrical cell arrangement under 97 % H_2_+3 % H_2_O between 0.1 and 1 MHz. Ag paint was used as a current collector in both cases.

## Supporting information

As a service to our authors and readers, this journal provides supporting information supplied by the authors. Such materials are peer reviewed and may be re‐organized for online delivery, but are not copy‐edited or typeset. Technical support issues arising from supporting information (other than missing files) should be addressed to the authors.

Supplementary

Supplementary
