# Supplementary material for: New Method for the Deposition of Nickel Oxide in Porous Scaffolds for Electrodes in Solid Oxide Fuel Cells and Electrolyzers
Source: ChemSusChem. 2016 Oct 14;10(1):258–65. doi: 10.1002/cssc.201600813 (PMC5248633; doi:10.1002/cssc.201600813)
Supplement: Supplementary file 2 — Supplementary [file CSSC-10-258-s002.pdf]

CHEMISTRY & SUSTAINABILITY
CHEMSUSCHEM
ENERGY & MATERIALS

Supporting Information

New Method for the Deposition of Nickel Oxide in Porous Scaffolds for Electrodes in Solid Oxide Fuel Cells and Electrolyzers

Enrique Ruiz-Trejo,* Milla Puolamaa, Brian Sum, Farid Tariq, Vladimir Yufit, and Nigel P. Brandon[a]

cssc_201600813_sm_IQM_analysis_Pores_BCYSZ_FT_Video.mp4
